# Immunohistochemical Analysis to Evaluate the Efficacy of Tetracycline-Loaded Nano-Chitosan in Treating Periodontitis Induced by *Porphyromonas gingivalis* in Albino Rats

**DOI:** 10.1155/ijod/1959086

**Published:** 2025-07-14

**Authors:** Mashael Saeed Alqahtani, Rania Hanafi Mahmoud Said

**Affiliations:** ^1^Division of Oral and Maxillofacial Pathology, Department of Basic and Clinical Oral Sciences, College of Dental Medicine, Umm Al-Qura University, Makkah, Saudi Arabia; ^2^Oral Pathology Department, Faculty of Dentistry, Suez Canal University, Ismaellia, Egypt

**Keywords:** dentistry, gingipains, immunohistochemistry, interleukin-1β, nano-chitosan, periodontitis, periodontology, *Porphyromonas gingivalis*, tetracycline

## Abstract

**Aim:** In this study, we examined the efficacy of tetracycline (TC) loaded on nano-chitosan in alleviating *Porphyromonas gingivalis*-induced periodontitis in albino rats. The location of the proinflammatory cytokine interleukin-1beta (IL-1β) was examined via immunohistochemistry.

**Methods:** Mature Wistar albino rats (*n* = 30; 150–180 g) were used in this study. The samples were split into five equal groups at random. The rats in all groups were subjected to antibiotics (for 3 days) in drinking water 1 week before the experiment started. Group I, which served as a control group, consisted of six albino rats. All groups except the control group were subjected to *P. gingivalis* injection by micro-pipetting 3 days weekly after ligation of the upper first molar (M1) for 4 weeks. Group II consisted of six rats that were injected with *P. gingivalis* twice weekly for 30 days. Six rats were assigned to Group III and subjected to the same treatments as those in Group II. However, in Group III, the infected regions were also injected with diluted TC powder daily for 2 weeks. Group IV was subjected to the same procedure as Group II before daily injections of nano-chitosan at the injection sites for 2 weeks. Group V consisted of six rats; once daily, they were administered localized injections of TC loaded on nano-chitosan for 2 weeks after the same procedure as Group II. After the rats were anesthetized, the maxilla, including the teeth and surrounding alveolar bone, was dissected. Light microscopy was performed for histological and immunohistochemical examinations of the biopsies. To check the normal distribution of samples Shapiro–Wilk test was performed, to determine the differences between the groups one-way analysis of variance (ANOVA) test was used, and to compare the groups pairwise, a Bonferroni post hoc correction was carried out. The results were statistically significant when *p* ≤ 0.05.

**Results:** Histological examination revealed that the periodontal tissues in Group I were normal. An increase in inflammatory cells (plasma cells, lymphocytes, and macrophages) was found in Group II, with alveolar bone resorption. A decrease in the number of dilated blood vessels and resorption areas was found in Group III. Inflammation and resorption in alveolar bone persisted in Group IV. In Group V, few dilated blood vessels were observed, with a decrease in inflammatory cells and alveolar bone deposition. Immunohistochemical examination revealed a significant difference in the level of IL-1β in the periodontal ligaments and alveolar bone between the negative control group and all other groups; however, the level of IL-1β in the alveolar bone was not significantly different between Groups II, III, and IV, whereas a significant difference in the level of IL-1β was detected between the different groups of periodontal ligaments.

**Conclusion:** Nano-chitosan may combat increasing TC resistance, with a noticeable enhancing effect on periodontal tissues infected with *P. gingivalis*.

## 1. Introduction

The tetracycline (TC) family is effective against many different types of gram-negative bacteria [1]. These compounds inhibit collagenase and inflammation, two key players in periodontitis therapy. Antibiotics belonging to the TC class prevent aminoacyl-tRNA from binding to the A site of the ribosomal complex [2]. This inhibition decreases protein synthesis in gram-positive and gram-negative bacteria. Antimicrobial resistance is a substantial worldwide public health concern in the 21^st^ century [3]. Several mechanisms can lead to TC drug resistance, including refluxing the drug before it reaches its target, avoiding the ribosomal binding area, modifying the permeability of the cell membrane, and preventing the uptake of drugs [4]. Many gram-positive and gram-negative bacteria are resistant to TC [5]. Antibiotic resistance is a serious concern as it has resulted in substantial monetary problems and human casualties. Over 700,000 people die every year because of antibiotic resistance, which is a result of the overuse of these drugs [6]. Periodontitis is caused by the oral microbial community's most prevalent pathogen, *Porphyromonas gingivalis* (*P. gingivalis*) [7]. The accumulation of bacteria in subgingival plaques provokes periodontitis. There are hundreds of periodontitis-causing bacteria in human subgingival plaque. Anaerobes of the mouth, *P. gingivalis*, are gram-negative bacteria [8]. According to the “Keystone Pathogen Hypothesis,” *P. gingivalis* can cause dysbiosis and disease by even modestly changing oral bacteria, which can lead to chronic periodontitis [9, 10]. Due to the availability of mutant strains with well-characterized genomes, many studies have been conducted on *P. gingivalis* [11]. Several virulence factors produced by *P. gingivalis* can act either alone or along with other mediators to cause inflammation [12]. When *P. gingivalis* is present, peripheral CD4^+^ T helper cells produce more proinflammatory cytokines, such as interleukin-1 and interleukin-6, which promote the development of severe periodontitis [13]. Many virulence factors, including structural components, are essential for the survival of *P. gingivalis* (heat shock proteins, fimbriae, and lipopolysaccharides), and secretory components (gingipains and outer membrane vesicles) are essential for survival and pathogenicity [4]. Nanoparticle (NP) therapy, nanostructured coatings of in vivo and other medical devices, and other similar approaches are being studied as methods for lowering antibiotic resistance and as nanodrug delivery systems. Particle structures with at least one dimension between 1 and 100 nm are known as NPs [14]. The surface area-to-volume ratio increases when the particle size decreases to the nanoscale, providing the NPs with a very high degree of flexibility, solubility, chemical reactions, and different morphologies with various modes of action [15]. Future cancer treatments may be used for developing NPs to deliver drugs since they can mitigate the harmful side effects of systemic medication administration and the damage caused by tumor excision [16]. Chitosan is made up of repeating units of N-acetylglucosamine (acetylated unit) and d-glucosamine (deacetylated unit), which are linked in a linear chain by β-1,4-glycosidic bonds. While most polysaccharides are either neutral or negatively charged under acidic conditions, the cationic nature of chitosan allows it to form electrostatic complexes with negatively charged synthetic or neutral polymers. Owing to its biocompatibility and biodegradability, chitosan is widely used in applications, such as protein delivery and wound healing [17]. Chitosan NPs are more effective than chitosan alone at fighting bacteria because of their unique physical and chemical properties. The higher surface charge density of the polycationic chitosan NPs helps them bind to the negatively charged surfaces of bacterial cells. This interaction causes the cell membranes of bacteria to break, releasing harmful contents [18]. First, chitosan is a harmless substance because it is biocompatible and biodegradable. Second, it is constructed from water-soluble polymers, which are advantageous for drug delivery carriers because they permit easy and gentle manufacturing processes. Therefore, chitosan NPs have several medical applications, including the delivery of macromolecules and unstable chemicals [19]. Third, the molecular weight of chitosan can be easily modified by coupling it with various ligands, which facilitates the development of novel products [20]. Moreover, chitosan shortens the time the substrate spends outside the cell, which increases the chances of absorption [21]. Owing to their small size, they improve drug transport across the cell membrane. The inclusion of NPs in the formulation facilitated their absorption and increased uptake of drugs [22]. Another advantage of chitosan NPs is that they can provide various distribution possibilities via noninvasive routes, such as through the mucosal linings of other canals [23]. Antimicrobial resistance is a growing public health concern due to the rapid appearance of new mechanisms of resistance. Some studies have highlighted the novelty of using TC-loaded chitosan NPs for periodontitis treatment. The combination of TC with nanocarriers, such as chitosan enhances drug stability, bioavailability, and controlled release, addressing some of the limitations of conventional treatments (e.g., poor tissue penetration and the need for frequent dosing) [24]. Additionally, the biocompatibility and ability of nano-chitosan to target specific sites within periodontal tissues further support its potential as a treatment strategy for periodontitis [25]. Therefore, in this study, we investigated how well TC loaded on chitosan NPs works to treat periodontitis caused by induced infection with *P. gingivalis* in albino rats. We aimed to determine the role that nanobiotechnology plays in overcoming TC resistance.

## 2. Null Hypothesis

TC loaded on nano-chitosan has no effect in treating induced periodontitis by *P. gingivalis* in albino rats.

## 3. Materials and Methods

The study protocol was approved by the Research Ethics Committee of the Faculty of Dentistry at Suez Canal University, Egypt (#356/2021). In every experiment, all principles and guidelines for the care and use of laboratory animals were strictly followed.

### 3.1. Sample Size Calculation

The sample size was calculated using G^*⁣*^*∗*^^Power version 3.1.9.2 [26]. The effect size convention f was 0.87 (large) according to previous studies with an alpha (*α*) level of 0.05 and a beta (*β*) level of 0.05, that is, power = 95%; the estimated sample size (*n*) revealed that at least 30 samples should be included and divided equally into five groups (six samples per group).

### 3.2. Sample Selection and Grouping

Adult Wistar albino rats (*n* = 30; weight: 150–180 g) were used in this study. Wistar albino rats were selected because they are less aggressive than other animal models, such as mice, have a short life span (about 2 years), and are slightly less expensive. The use of a “ligature” model of periodontitis in Wistar rats offers several advantages. These include a short disease induction period (30 days), noticeable clinical inflammation of periodontal tissues, and significant alveolar bone resorption [27]. The rats were kept in suitable numbered cages with access to running water and dry rat pellets in a properly ventilated animal facility at 27–30°C. The animals were monitored for changes in weight.

#### 3.2.1. Before Treatment

• One week before ligature placement, all rats (from all groups) received antibiotics (1 mg/mL sulfamethoxazole and 200 μg/mL trimethoprim) in their drinking water for 3 days. This treatment decreased the natural bacterial load in their mouths [28].

**Figure 1:**
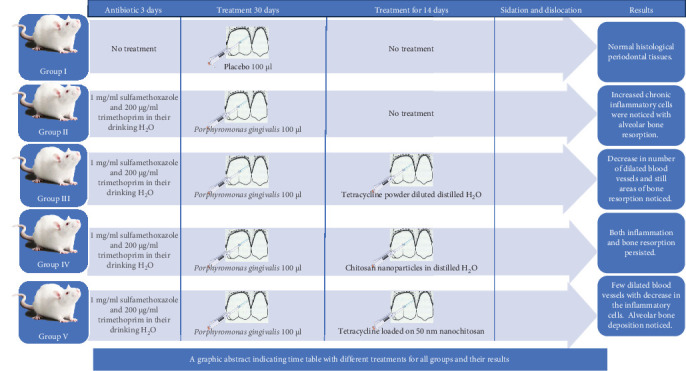
The graphic abstract shows different treatments of all groups and their results.

The albino rats were divided into the following five groups (Figure 1). Group I included six albino rats and served as a negative normal control group. Group II (infection control) contained six albino rats.

Ligature placement and bacterial exposure.

• For 30 days, a bacterial mixture (100 μL) was applied around the ligature on the first molar (M1) 3 days per week [29].

#### 3.2.2. Surgical Procedure

• Under general anesthesia, the circumference of the right upper first molar (M1) was tied with a special suture (3–0 silk), while the mouth was held open with a specific instrument (Hashimoto's gag). The suture knot was secured to the front of the M1 tooth via dental filling material [29].

#### 3.2.3. Bacterial Injection

• While anesthetized with isoflurane, the rats received an injection of 0.5 mL/kg of a solution containing *P. gingivalis* through the interdental gingiva using a micropipette with a sterile pointer between the first and second upper molars on both sides. A 1/2 McFarland optical density was used to alter the inoculum opacity, which corresponds to a concentration of 1 × 10^8^ CFU/mL (BD Franklin Lake, New Jersey, USA) [30].

As *P. gingivalis* infections are spread through simple contact with mucous membranes, spread and inoculation may occur accidentally. Therefore, the transmission of *P. gingivalis* can occur after an infected animal bites. Thus, the injection was managed under the supervision of a scientific expert at the laboratories of the National Research Center, Egypt, to ensure infection control instructions and decrease the risk of infection.

Group III (treated with TC) included six albino rats, which followed the same protocol as Group II before receiving a daily injection at the same infection site with 1/2 mL/kg TC powder diluted in 50 g/mL distilled water for 14 days [31].

Group IV included six rats that followed the same process as Group II before being injected daily with 1/2 mL/kg chitosan NPs dissolved in 50 g/mL distilled water at the same area of infection for 14 days [32].

Group V included six albino rats subjected to the same process as Group II before a daily injection of 1/2 mL/kg TC loaded with 50 nm nano-chitosan dissolved in distilled water at a concentration of 140 g/mL for 14 days.

After the treatments in Groups III, IV, and V ended (primary end point), all rats were sedated and killed by dislocation, which was the final step of the experiment. The maxillae of the rats were removed and fixed in 10% neutral buffered formalin overnight, after which 10% EDTA was used for decalcification, which required a week. After the tissues were processed, they were embedded in paraffin blocks. From the paraffin blocks, the tissues were cut into sections (4 μm), stained with hematoxylin and eosin, and subjected to immunohistochemical examination. Periodontal histological and inflammatory reactions were detected using a rat monoclonal antibody for immunohistochemical investigation of IL-1β. The avidin–biotin-peroxidase method was used.

### 3.3. Culturing *P. gingivalis*

*P. gingivalis* can thrive in a medium that contains tryptone soy agar broth (TSB), which should also contain 5% sheep blood, 1 g/L menadione, 1 g/L hemin, and 5 g/mL erythromycin. Anaerobic chamber (Coy) cultures were maintained with 5% CO_2_, 85%–55% nitrogen, and 10% hydrogen [33]. After the cells were centrifuged at 12,000 rpm for 5 min from the growth medium for the next 24 h, the cells were washed three times with sterile water. The cells were then resuspended in phosphate-buffered saline (PBS) and centrifuged at 12,000 rpm for 5 min. The supernatant was removed, and the cells were resuspended in PBS. The last step was repeated many times, and the optical density of *P. gingivalis* bacterial growth was subsequently measured using a UNIKONXL spectrophotometer (Northstar Scientific Ltd, TudorHouse, 1A–1B Barleyhill Road, Garforth, Leeds, LS25 1DX, United Kingdom) at 600 nm (OD600). To measure the bacterial growth, the McFarland test was used, which involves suspending newly grown bacteria in a solution at a concentration of 1 × 10^8^ CFU/mL relative to the 1/2 McFarland standard [30]. Culturing *P. gingivalis* was conducted in the Department of Microbiology in the Agriculture Faculty of the Egyptian National Research Centre.

### 3.4. Characterization and Preparation of Chitosan NPs

Chitosan NPs loaded with TC were obtained from Cairo, Egypt, by the Nano-Gate Company. The ionotropic gelation approach is used to create chitosan NPs by cross-linking the positively charged amino groups of chitosan with the negatively charged anions of tri-polyphosphate (TPP). In the first step, 28.7 mL of 1% v/v acetic acid, containing 2 mg/mL or 0.5 mg/mL of chitosan powder, was mixed, and the mixture was stirred for 2 h to make a homogeneous solution. In the second step, the solution was filtered to remove impurities. In the third step, a water-based TPP solution was slowly added to a chitosan solution. In the fourth step, after the complete addition of TPP, the turbid solution was sonicated for 30 min, centrifuged with DH_2_O at 15,000 rpm, and finally, it was frozen and dried [34]. To create chitosan NPs encapsulated with TC, TC was liquefied in a chitosan solution. Then, TPP was added very slowly to the solution with magnetic stirring to avoid the photodegradation of TC, so it is preferable to be delivered at night [35].

**Figure 2:**
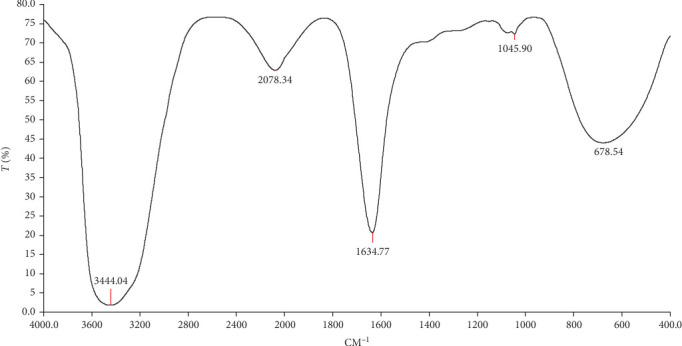
The FTIR spectra of Tetra@ChNPs display their wavelength (cm^–1^) versus transmittance (%).

**Figure 3:**
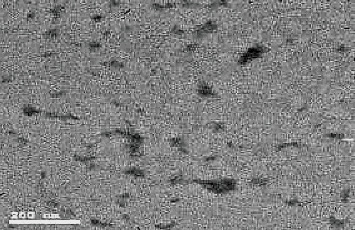
The primary nanoparticles of chitosan are 30–50 nm in size and are spherical, as examined by spectral signature identification at 430 nm.

A spectral study was conducted to develop NPs under various reaction conditions, and 24 peaks were obtained. Fourier transform infrared spectroscopy (FTIR), Bruker Corporation, Billerica, Massachusetts, USA, was used to examine the nano-chitosan spectra (Figure 2). A transmission electron microscope (TEM; JEM1200EX; JEOL Ltd, Akishima, Tokyo 196-8558, Japan) was used to analyze the configuration of the primary chitosan NPs, including their size and shape (Figure 3).

To ensure the validity of the data collection, the outcome of the study was evaluated by a trained and calibrated pathologist who was blinded to the samples and groups.

### 3.5. Detection of IL-1β Immunohistochemically

The detection method for rat tissues was as follows: HRP/DAB was mixed with the primary antibody, which is a monoclonal antibody against rat IL-1β. An immune-enzymatic marker, namely, a streptavidin-biotin antigen detection system, is one of the reagents included in the kit. The primary antibody (anti-IL-1β monoclonal antibody [EPR24895-116], Abcam Co., Cambridge, United Kingdom) was combined with antirat HRP/DAB (ab236466). The reagents used in the kit consisted of an immune-enzymatic marker (a streptavidin-biotin antigen detection system). After adding an antigen-specific unconjugated primary antibody, a biotinylated secondary antibody that reacts with the primary antibody, an enzyme-labeled streptavidin-biotin system (LSAB 2 System-HRP, K0609; Dako Inc., Carpinteria, CA, USA), and a chromogen (DAB) were added to the slide section in a precise order. All evaluations were conducted in a blinded manner. For each group, 100 cells were counted at 40x magnification to calculate the percentage of marker-positive cells within the designated areas. Image analysis was performed using the ImageJ software (version 1.48; National Institutes of Health [NIH], Bethesda, Maryland, USA). The data obtained from the image analysis were statistically analyzed. Morphometric assessments were conducted using the cellSens Life Science Imaging Software, Olympus Co., Hachioji, Tokyo, Japan. The degree of the intensity of IL-1β immunostaining was estimated by analyzing the degree of mild, moderate, and strong DAB brown staining [36].

The Microbiologics company transported [37] strains of *P. gingivalis* ATCC 33277. *P. gingivalis* was delyophilized and cultured at the Egyptian National Research Centre.

### 3.6. Statistical Analysis

All data were calculated, tabulated, and statistically analyzed by conducting the following statistical tests. A normality test (Shapiro–Wilk), IBM Co, NY, USA, was performed to check the normal distribution of the samples. Descriptive statistics were calculated as the mean ± standard deviation (SD). One-way analysis of variance (ANOVA), IBM Co, NY, USA, was performed to determine differences between the groups. A Bonferroni post hoc, IBM Co, NY, USA, correction was performed for pairwise comparisons among the groups. All results were considered to be statistically significant at *p* ≤ 0.05. Statistical analysis was performed using SPSS version 26.0 (Statistical Package for Social Science, Armonk, NY: IBM Corp.) for Windows at *p*  < 0.05 (significance level).

## 4. Results

### 4.1. Characterization of the NPs

The size and shape of the chitosan NPs were measured. Electron microscope photographs revealed that the primary particle size ranged from 30 to 50 nm and was mostly spherical, as determined by spectral signature identification at 430 nm.

The characterization and preparation of chitosan NPs were described in a study by Taha and Said [38].

### 4.2. Histopathologic Results

**Figure 4:**
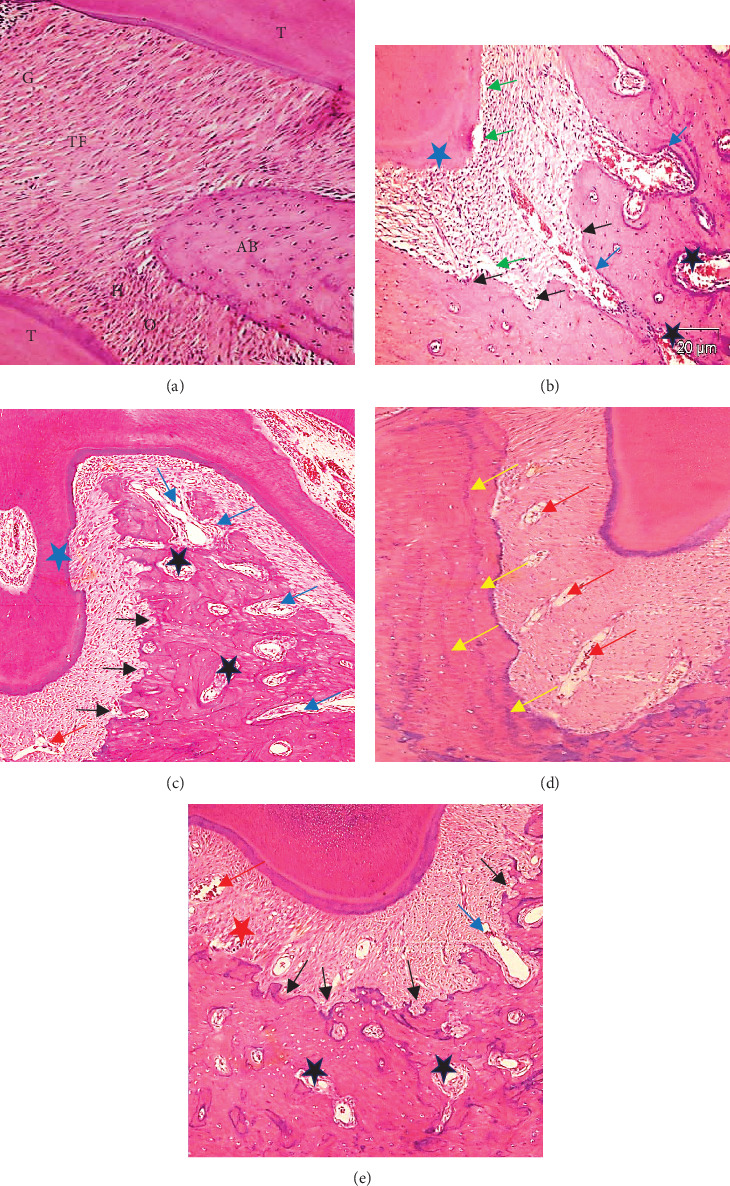
(A) Group I. Normal periodontal tissue structures, gingival periodontal ligament (G), transeptal fibers (TF), alveolar crest fibers (AC), horizontal fibers (H), oblique fibers (O), interdental alveolar bone (AB), and teeth (T) (H&E. orig. mag. ×400). (B) Group II (infection group). (C) Group III (treated with TC). (D) Group IV (treated with chitosan nanoparticles). (E) Group V (treated with TC loaded on chitosan nanoparticles). Red arrows show dilated blood vessels. Green arrows show the detachment of periodontal ligament fibers. Black arrows show alveolar bone resorption. Black stars refer to marrow cavities with fibrotic content. Blue arrows show the widening of the Zuckerkandl and Hirschfeld canals. Blue stars refer to cementum resorption. Red stars refer to localized areas of fiber dissociation. Yellow arrows show reversal incremental lines. (H&E. orig. mag. ×250).

The periodontium in Group I (the control group) had normal histological characteristics of nerves, blood vessels, fibers, and cells under a light microscope. The periodontal ligaments showed normal principal fibers, the gingival group of fibers connected to the cervical portion of the cementum and extended to the free and attached gingiva, where they fuzed with the gingival lamina propria and collagen fibers that make up most of the PDL. The fibers in the interdental or trans-septal group extended above the crest of the alveolar bone from the cementum of one tooth to the cementum of the neighboring tooth. The alveolar crest fibers, as well as the horizontal, oblique, apical, and interradicular fibers, constitute the alveolo-dental group of fibers that are linked to the cementum and the alveolar bone. The fibers were anchored from both sides, the tooth cementum and the alveolar bone. The bone and cementum surfaces presented normal architectures with no signs of resorption (Figure 4A).

Group II (infection group): Periodontium examination of Group II rats injected with *P. gingivalis* bacteria revealed detachment and dissociation of principal fibers with inflammatory cell infiltration (plasma cells, lymphocytes, and macrophages), noticeably enlarged blood vessels, several Howship's lacunae and osteoclasts on the bone surface, widened marrow cavities with increased inflammatory cell and RBC numbers, and widened Zuckerkandl and Hirschfeld canals. Areas of cementum resorption were recorded (Figure 4B).

Group III (treated with TC): The periodontal ligaments were mostly organized, with notable decreases in inflammatory cells (plasma cells, lymphocytes, and macrophages), some dilated blood vessels, and no detachment from either the bone or teeth. Multiple areas of bone resorption widening were found by Zuckerkandl and Hirschfeld. The bone marrow cavities widened as the fibrotic content increased. Areas of cementum resorption were recorded (Figure 4C).

In Group IV, the patients in the chitosan-treated group had a poor prognosis, and the periodontal ligaments presented with vasodilated blood vessels, inflammatory cell infiltration (plasma cells, lymphocytes, and macrophages), localized areas of fiber dissociation and detachment from both the bone and cementum sides, and widening of the Zuckerkandl and Hirschfeld canals. Fibrotic content of bone marrow cavities was obvious. Multiple areas of alveolar bone resorption appeared mostly around the bone surface (Figure 4D).

Group V (TC-chitosan NP-treated group): The periodontal ligaments appeared organized with low-grade inflammatory cell infiltration (plasma cells, lymphocytes, and macrophages), few dilated blood vessels, and a localized area of fiber detachment from the bone side. The alveolar bone surface exhibited a normal appearance, with many reversal lines indicating osteoclastic activity followed by bone deposition on the bone surface. The number of inflammatory cells in all periodontal tissues decreased, but few blood vessels in the periodontal ligament remained prominent (Figure 4E).

### 4.3. Immunohistochemical Results

**Figure 5:**
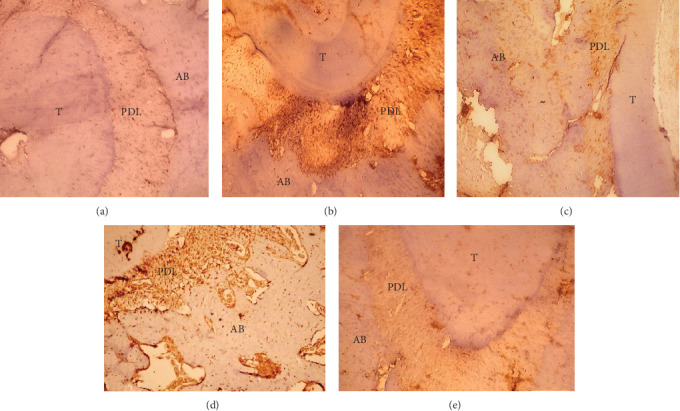
(A) The control group reacted negatively to IL-1β. (B) Group II reacted strongly positively to IL-1β in the periodontal tissue and alveolar bone. (C) Group III reacted mildly to IL-1β through the periodontal tissue and alveolar bone. (D) In Group IV, the periodontal tissues and alveolar bone reacted moderately positively to IL-1β. (E) In Group V, the reactivity to IL-1β was very mild in the periodontal tissues and negative in the alveolar bone. (T) Tooth, (AB) alveolar bone, and (PDL) periodontal ligaments. (IL-1β antibody origin. mag. ×250).

Immunohistochemical examination of IL-1β: different immune responses to IL-1β occur in periodontal tissues and alveolar bone. Group II exhibited a strong positive response to IL-1β in periodontal tissues and alveolar bone. In Group III, a mild positive reaction was found in periodontal tissue and alveolar bone, whereas the TC-treated group exhibited a mild positive reaction, particularly in periodontal tissue. Group IV exhibited a moderately positive response to IL-1β in the alveolar bone and PDL fibers. In Group V, periodontal tissues treated with TC-chitosan NPs presented a very mild positive response to IL-1β in the periodontal tissue and a negative response in the alveolar bone (Figure 5).

### 4.4. Immunohistochemical Analysis of IL-1β

Analysis of the data presented in Tables 1 and 2 revealed a significant disparity in the mean IL-1β optical density of the studied groups, as determined by one-way ANOVA (*F* = 5825.16, *p*=0.0001 for periodontal ligaments and *F* = 1651.94, *p*=0.0001 for alveolar bone). Pairwise comparison showed a significant difference in the level of IL-1β in the periodontal ligaments and alveolar bone between the negative control group and all other groups; however, the level of IL-1β in the alveolar bone was not significantly different between Groups II, III, and IV, whereas a significant difference in the level of IL-1β was found between the different groups of periodontal ligaments (Tables 1 and 2).

## 5. Discussion

Inflammation of the gingivae, periodontal ligament, alveolar bone, cementum, and all of the supporting structures of the teeth accompanies periodontitis [36]. These methods can cause teeth to exfoliate in severe cases [36]. These diseases are common in the human population. The American Academy of Periodontology (AAP) divides periodontal diseases into two main groups based on how much periodontal tissue is affected: gingivitis and periodontitis [39]. The main cause of gingivitis is the accumulation of dental plaque, which causes inflammation in the long term. Clinically, the gingivae appear swollen with redness and easily bleed [40]. Since the periodontal ligament takes some time to become involved in the inflammatory process, it may not be affected immediately by gingivitis. In this study, albino rats were selected as the model for periodontitis because of their well-documented susceptibility to experimental periodontitis when periodontitis is induced through mechanical or microbial means [41]. Additionally, the availability of established protocols for inducing periodontitis in albino rats, along with the similarity in inflammatory and bone resorption processes to human periodontitis [42], makes them a suitable model for testing new therapeutic techniques, such as TC-loaded nano-chitosan. This choice is supported by other studies that have used albino rats in periodontal research and reported promising results [43, 44]. The results of this study showed that chronic periodontitis appears in ligated areas 30 days after infection with *P. gingivitis*, which matches the findings of de Molon et al. [31]. Periodontal tissue was selected for this investigation because, if ignored, it can lead to very harmful effects on the alveolar bone and the periodontal ligament that may lead to tooth loss. Additionally, IL-1β is a proinflammatory cytokine that promotes alveolar bone resorption in periodontitis through the induction of cellular proteinases [45, 46]. IL-1β functions in periodontitis in three collaborative ways, first by increasing the activity of enzymes that break down tissues. IL-1β stimulates the production of matrix metalloproteinases (MMPs), particularly MMP-9, which degrade collagen and other components of the extracellular matrix (ECM) surrounding teeth. Second, by promoting bone breakdown. It directly increases the production of receptor activator of NF-κB (RANKL), a molecule that activates osteoclasts. It also decreases the production of osteoprotegerin (OPG), a natural inhibitor of RANKL, further promoting bone resorption [47]. Third, it indirectly influences bone loss: it triggers the release of other inflammatory molecules, such as prostaglandin (PGE_2_) and CX3CL1, which further contribute to osteoclast activity and bone breakdown [48]. Periodontitis in this experimental model is driven primarily by *P. gingivalis*, a keystone pathogen recognized for its potent virulence factors, including gingipains, which critically disrupt host immune responses. The presence of *P. gingivalis* initiates a dysbiotic shift in the subgingival microbiota, leading to a greater inflammatory response. This inflammation promotes the release of proinflammatory cytokines, such as IL-1β, TNF-α, and IL-6, which in turn increase the expression of RANKL. The therapeutic intervention evaluated in this model not only targets the bacterial component but also modulates the RANKL/OPG balance. An intervention that effectively inhibits *P. gingivalis* virulence factors may also prevent excessive activation of osteoclasts, thereby reducing the subsequent bone resorption observed in periodontitis. Histological assessments, such as TRAP staining, can provide further validation of reduced osteoclast numbers in treatment groups [49]. As IL-1β levels increase in periodontitis and increase over time, it is used as an immunohistochemical marker, and its increase indicates the propagation of periodontitis.

The results of this study are similar to those of Group II, which showed a strong positive reaction against IL-1β, indicating considerable destruction of periodontal tissues by *P. gingivalis*. This pathogen increases the level of IL-1β in periodontal tissues, as revealed by alveolar bone resorption. In Group II, where the periodontal connective tissue of the rats was infected with *P. gingivalis*, a high number of inflammatory cells (plasma cells, lymphocytes, and macrophages) were present, which was explained by Ji and Choi [50], as *P. gingivalis* is essential for the separation of collagen fibers and connective tissues. *P. gingivalis* can enter subepithelial connective tissue before cell rupture when it infiltrates and multiplies inside epithelial cells. This pathogen uses epithelial cells as vectors, which enables them to spread after desquamating the oral mucosa lining and then spread to the underlying periodontal tissues [50]. Dipeptidyl aminopeptidase IV (DPPIV) is a powerful factor produced by *P. gingivalis* that contributes to the degradation of connective tissue. DPPIV plays an important role in the pathogenicity of *P. gingivalis*. Periodontitis caused by *P. gingivalis* progresses when the bacteria invade connective tissues, mostly through paracellular pathways, and attach to the ECM through the interaction of the colonizing proteins DPPIV and fibronectin. Many proinflammatory mediators are produced after infection with a bacterium, which attracts inflammatory cells (which may not be present in large numbers) [51]. Moreover, *P. gingivalis* activates MMPs by releasing numerous proteases, such as DPPIV and gingipains. The long-term consequence of bacteria producing DPPIV, gingipains, and MMPs is damage to ECM proteins. Inflammation causes fibroblasts to adhere less strongly to fibronectin, and DPPIV also decreases their mobility and attachment to ECM components [51, 52]. Additionally, *P. gingivalis* is associated with an aberrant host response, which implies that persistent hyperinflammatory disorders may develop if the inflammatory response is not adequately controlled in such a complicated setting [51]. Pattern recognition receptor (PRR) stimulation by pathogen-associated molecular patterns (PAMPs) is initiated by tissue damage-induced persistent hyperinflammatory responses. Epithelial, neutrophilic, macrophagic, and dendritic cells have PRRs on their surfaces [52]. By stimulating the innate response to microbial attack and, by extension, generating adaptive immunity to eradicate infections, PRRs activated by PAMPs can activate PRRs. Tissue damage-induced persistent hyperinflammatory responses involve PRR stimulation by PAMPs [51]. Individual lymphocytes are present, along with masses of dendritic cells and lymphoid follicles, in oral mucosa immunity. The most significant types of dendritic cells in the oral mucosa are Langerhans cells, macrophages, and mast cells [53]. The activation and maturity of dendritic cells influence their ability to stimulate immune responses. To bring in additional immune cells, such as monocytes, T cells, and natural killer cells, active dendritic cells release chemokines [54]. *P. gingivalis* secretes gingipains, which are trypsin-like proteinases. They play a key role in the pathogenesis of periodontitis by degrading host proteins, maturing fimbriae, and promoting sustained bacterial colonization. Inhibiting gingipains through vaccination or specific inhibitors can attenuate *P. gingivalis*-induced disorders. HRgpA and RgpB are key gingipains that induce vascular permeability and activate the blood coagulation system, contributing to inflammation and bone loss in periodontitis. Kgp, another gingipain, is involved in fibrinogen degradation and bleeding tendencies in diseased gingiva. Targeting gingipains may be an effective way to treat periodontal disease [55]. Besides being effective against gram-positive and gram-negative bacteria, TCs are also versatile antibiotics that can be taken orally or administered intravenously (IV) as injections. Clinical studies have shown that TC is safe and well-tolerated. The antibacterial properties of TC are susceptible to intrinsic antibiotic resistance mechanisms [56]. Fernandes et al. [57]investigated subgingival irrigation with TC hydrochloride (TTC-HCL) as a treatment for induced periodontal disease in rats. The results showed that the group receiving subgingival irrigation with TTC-HCL presented less bone loss than the control group at 7, 15, and 30 days after treatment. These findings suggested that subgingival irrigation with TTC-HCL is an effective adjunctive treatment for periodontal disease in rats [57]. These results were similar to those of this study. In group III, injection of TC for 14 days after infection with *P. gingivalis* for 30 days decreased inflammation. Although the use of TC injection is very effective, some areas of alveolar bone resorption still appear. This may be explained by the fact that an insufficient amount of TC can stimulate PGE_2_ and CX3CL1; these, in turn, stimulate osteoclasts, which are responsible for alveolar bone resorption [47].

In Group IV, both inflammation and bone resorption persisted, as demonstrated by a strong positive reaction against the IL-1β antigen. These results were similar to those reported by Taha and Said [38]. They found that nano-chitosan had some antimicrobial activity against *P. gingivalis*, but its effect was weak [38]. The weak anti-inflammatory effect of using nano-chitosan alone as an autograft in the calvarium cavities of rabbits was demonstrated by Jafarzadeh et al. [58]. The results of these previous studies suggested that low doses of nano-chitosan have weak anti-inflammatory effects. Ardila et al. [59] reported an increase in bacterial resistance against TC despite a decrease in the use of TC in recent years. They owed that resistance to genetic mutations in microorganisms found in the subgingival area. When homeostasis in subgingival areas is disturbed, the immune system starts healing [59]. To overcome the growing bacterial resistance against TC and obtain the optimum effects of TC as a potent broad-spectrum antibiotic, in this study, TC loaded on nano-chitosan was used after inducing infection of periodontal tissues with *P. gingivalis*. In Group V, the use of TC loaded with nano-chitosan had strong positive effects on periodontal tissues, as it decreased the inflammatory response triggered by *P. gingivalis* infection. TC antibiotics possess immunomodulatory effects along with their antimicrobial properties, strongly enhancing the antigen-presenting function of Langerhans cells, which is vital for initiating adaptive immune responses [60]. Langerhans cells, which are found in the skin and mucosal epithelia, detect pathogens and antigens, process and present them to T-cells in nearby lymph nodes, which is crucial for immune activation. TC enhances the functions of Langerhans cells by inhibiting MMPs, preventing ECM protein degradation, exerting anti-inflammatory effects, reducing local inflammation, and promoting optimal antigen presentation. Additionally, TC modulates intracellular signaling pathways, such as the NF-κB and MAPK pathways, regulating the expression of genes involved in antigen presentation and immune activation in Langerhans cells [61]. TC increases the ability of Langerhans cells to present antigens, which implies the role of TC in the regulatory function of the immune system [62]. Dermal keratinocytes of CD301b-expressing mice, which are known as macrophage galactose-type C-type lectin-2 (MGL-2), decrease the stimulation of T-helper cell reactions via interferon regulating factor-4 (IRF-4). Tissue regeneration was promoted by increasing the formation of granulation tissue and enhancing the amount of regenerated periodontal tissue [62].

NPs need to be safe for use by the human body; thus, they should break down naturally and not cause any kind of immunological response. In recent years, many studies have investigated NPs and their possible use as antibacterial agents and delivery methods for antibiotics. Using nanocarriers in this setting has several advantages, such as better biodistribution, greater drug solubility, and a lower dosage tolerance. Methods for the targeted and long-term distribution of pharmaceuticals include controlling their cotransport and prolonged circulation in the bloodstream. Many studies have used different types of nanocarriers that meet all desirable criteria and provide the optimum biological targets [63]. Due to its NH_2_ group structure, chitosan, when used as an NP carrier, may function in acidic environments. Chitosan NPs, which are positively charged, can bond with other materials that are negatively charged via cross-bonding [64]. Cross-bonding is highly beneficial, both physically and chemically, as it decreases the adverse effects on the body. As nano-chitosan has many amino groups, vehicles with greater surface areas may be produced [65]. Amalia et al. [66] reported that chitosan NPs loaded with 0.7% TC effectively inhibited *P. gingivalis* growth in vitro, as indicated by a zone of inhibition of 32.8 mm. In an in vivo study in 2023, the potential effects of TC loaded on nano-chitosan on gingivitis and its ability to decrease the harmful effects of the bacterium *P. gingivalis*, a key player in gingivitis and periodontitis initiation, were investigated. The results showed that TC loaded on nano-chitosan was more effective against *P. gingivalis*. TC loaded on nano-chitosan can promote the growth of fibroblasts and the formation of granulation tissue, which are important for wound healing. This finding matched the results of our study, where Group V rats treated with TC loaded on nano-chitosan presented better fibroblast viability than those receiving TC alone (Group III) [38].

A study conducted in 2011 revealed that TC can prevent *P. gingivalis* from growing by decreasing the content of IL-1β and TNF produced by host monocytes. Therefore, in this study, antibodies against IL-1β were expressed mildly in the rats in group III and only very mildly in the rats in Group V. The rats in group III received only TC, while the rats in Group V received TC-chitosan NPs; the results indicated that TC-chitosan has a stronger effect on *P. gingivalis* than TC alone, which was interpreted as the lack of a significant difference between Groups V and I [67].

The results of this study revealed that the immune expression of IL-1β was milder in the superficial layers than in the basal cell layers. Some immune expression was found in the lamina propria, blood vessels, and connective tissue fibers. In Groups III and V, the number of inflammatory cells in the lamina propria, including plasma cells, lymphocytes, and macrophages, was lower than that in Group II. Such a pattern occurred probably because inhibiting prostaglandin E_2_ synthesis via phospholipase A_2_ and the anti-inflammatory properties of nano-TCs can reduce the activity of polymorphonuclear cells (PMNLs), as demonstrated by the results of Group V. Nano-TCs can reduce neutrophil-mediated tissue damage by inhibiting neutrophil migration and degranulation, along with decreasing oxygen radical synthesis [68]. Moreover, it could be interesting to test TC-loaded nano-chitosan therapy in combination with other adjunctive treatments, such as ozone, photobiomodulation, and paraprobiotics in order to understand their mutual effect on periodontal tissues [69–71].

## 6. Limitations

Although this study demonstrated promising therapeutic effects of TC-loaded nano-chitosan in treating *P. gingivalis*-induced periodontitis in a rat model, it had several limitations. First, while the rat model effectively mimics the inflammatory and bone resorption features of periodontitis, it does not fully represent the complexity of the human periodontal disease. Differences in the immune response, oral microbiota composition, and healing dynamics between rats and humans may affect the translational relevance of the findings. Second, the study focused on a single dose and concentration of TC-loaded NPs. However, the optimal therapeutic dose in humans may vary due to differences in drug absorption, local tissue conditions, and disease severity among individuals. Dose-response studies are necessary to fine-tune treatment protocols for clinical application. Finally, while *P. gingivalis* is a key pathogen in periodontitis, and the disease is polymicrobial. Targeting a single organism may not fully address the broader microbial imbalance and inflammatory processes in the human periodontal environment. Future studies should include long-term evaluations, different dosing regimens, and trials in larger animal models or clinical settings to better assess efficacy and safety.

## 7. Conclusion

Coating chitosan NPs with TC may be a promising strategy to prevent antibiotic side effects and combat TC resistance. By combining the antibacterial properties of TC with the biocompatibility and sustained-release capabilities of nano-chitosan, this formulation offers a targeted approach to controlling periodontal inflammation and limiting tissue destruction. The observed reduction in IL-1β expression and signs of tissue healing in the experimental model suggested that this strategy can effectively suppress pathogenic activity while promoting periodontal regeneration. With further optimization and validation through dose-ranging studies and clinical trials, this therapeutic approach may serve as a valuable alternative to conventional periodontal treatment, especially in localized, nonsurgical applications where sustained drug delivery is beneficial.

## 8. Ethical Statements for “HUMAN and ANIMAL RIGHTS”

This study adhered to internationally accepted standards for animal research, following the “3R” principle. The Animal Research: Reporting of In Vivo Experiments (ARRIVE) guidelines were followed while conducting experiments involving live animals, promoting ethical research practices.

## 9. Recommendations

The mechanical stress caused by injecting saline into tissues warrants further research with larger samples and an additional positive control group. Additionally, more data on the release kinetics of TC from chitosan NPs are needed to accurately account for the amount of drug released into the tissues. More studies are needed to investigate the role of other inflammatory cytokines (besides IL-1β), such as TNF-α, IL-6, RANKL, and OPG, in the inflammatory reaction of induced periodontitis and subsequent alveolar bone resorption.

## Figures and Tables

**Table 1:** Differences in the mean IL-1β optical density in periodontal ligaments among the different groups.

IL-1β for periodontal ligaments								
Groups			Mean	SD	CI	*F* test	*p*-Value	
Control (G1)			51.9^e^	2.9	46.22, 57.58	5825.16	0.0001^*∗*^	
Infected (G2)			225.9^a^	2.3	221.39, 230.41			
Tetracycline (G3)			205.8^c^	2.6	200.70, 210.90			
Chitosan (G4)			208.9^b^	1.8	205.37, 212.43			
Tetracycline loaded on chitosan (G5)			118.2^d^	1.6	115.06, 121.34			

**Pairwise comparison using Bonferroni post hoc**								
**Compared groups**	***p*-Value**		***p*-Value**			***p*-Value**		***p*-Value**

G1 vs. G2	0.0001^*∗*^	G2 vs. G3	0.001^*∗*^	G3 vs. G4		0.001^*∗*^	G4 vs. G5	0.001^*∗*^
G1 vs. G3	0.0001^*∗*^	G2 vs. G4	0.001^*∗*^	G3 vs. G5		0.04^*∗*^	—	—
G1 vs. G4	0.0001^*∗*^	G2 vs. G5	0.001^*∗*^	—		—	—	—
G1 vs. G5	0.001^*∗*^	—	—	—		—	—	—

*⁣* ^*∗*^ and different superscript letters indicate significant difference between groups at *p*  < 0.05.

**Table 2:** Differences in the mean IL-1β optical density in the alveolar bone between the different groups.

IL-1β for Alveolar bone									
Groups			Mean	SD	CI	*F* test	*p*-Value		
Control (G1)			41.2^c^	2.9	35.52, 46.88	1651.94	0.0001^*∗*^		
Infected (G2)			146.3^a^	3.9	138.66, 153.94				
Tetracycline (G3)			147.9^a^	3.5	141.04, 154.76				
Chitosan (G4)			146.6^a^	2.7	141.31, 151.89				
Tetracycline loaded on chitosan (G5)			60.5^b^	2.9	54.82, 66.18				

**Pairwise comparison using Bonferroni post hoc**									
**Compared groups**	***p*-Value**		***p*-Value**			***p*-Value**			***p*-Value**

G1 vs. G2	0.0001^*∗*^	G2 vs. G3	0.947	G3 vs. G4		0.851		G4 vs. G5	0.0001^*∗*^
G1 vs. G3	0.0001^*∗*^	G2 vs. G4	0.861	G3 vs. G5		0.0001^*∗*^		—	—
G1 vs. G4	0.0001^*∗*^	G2 vs. G5	0.0001^*∗*^	—		—		—	—
G1 vs. G5	0.0001^*∗*^	—	—	—		—		—	—

*⁣* ^*∗*^ and different superscript letters indicate significant difference between groups at *p*  < 0.05.

## Data Availability

The data supporting the findings of this study are openly available at and can be accessed at https://doi.org/10.4103/jpbs.jpbs_532_22. This includes raw data from immunohistochemical analyses, experimental results, and supporting information.
